# Smaller and thinner long bones in children and adolescents with cerebral palsy and other neuromotor impairments

**DOI:** 10.3389/fendo.2025.1620573

**Published:** 2025-06-30

**Authors:** Erin Hodgson, Elizabeth G. Condliffe, Leigh Gabel

**Affiliations:** ^1^ Department of Biomedical Engineering, University of Calgary, Calgary, AB, Canada; ^2^ Faculty of Kinesiology, University of Calgary, Calgary, AB, Canada; ^3^ McCaig Institute for Bone and Joint Health, University of Calgary, Calgary, AB, Canada; ^4^ Alberta Children’s Hospital Research Institute, University of Calgary, Calgary, AB, Canada; ^5^ Departments of Clinical Neurosciences and Pediatrics, University of Calgary, Calgary, AB, Canada; ^6^ Hotchkiss Brain Institute, University of Calgary, Calgary, AB, Canada

**Keywords:** cerebral palsy, bone health, muscle health, neuromotor impairments, children, adolescents, pQCT

## Abstract

**Introduction/Background:**

Compromised bone and muscle health is a significant concern for children and youth with cerebral palsy (CP) and other non-progressive neuromotor impairments. Weak bones increase the incidence of fragility fractures and predispose individuals to lifelong problems, such as osteoporosis.

**Objectives:**

This study quantified bone and muscle health in children and adolescents with CP and other neuromotor impairments across all five gross motor function classification system (GMFCS) levels.

**Methods:**

Peripheral quantitative computed tomography (pQCT) scans of both tibiae were acquired at the 3%, 38%, and 66% of tibia length in 22 children and adolescents (4–17 years old) diagnosed with CP and “CP-like” neurodevelopmental conditions causing motor impairment. Age-, sex-, and ethnicity-matched Z-scores were generated in reference to a normative typically developing population for total bone mineral content (BMC), trabecular and cortical bone mineral density (Tb.BMD, Ct.BMD), cortical BMC (Ct.BMC), cortical area (Ct.Ar), cortical thickness (Ct.Th), periosteal and endosteal circumference, cortical section modulus (Z), and muscle cross-sectional area (MCSA).

**Results:**

Tibial total BMC, Tb.BMD, Ct.BMC, Ct.Th, Ct.Ar, periosteal circumference, Z, and MCSA were significantly lower in children with CP and CP-like conditions compared to typically developing peers (median Z-scores ranged from -2.66 to -1.09; p = 0.019 to <0.001) and showed greater deficits in children and adolescents with lower levels of motor function than those with higher functional abilities (GMFCS I-II *vs* III-V; p = 0.042 to <0.001). Endosteal circumference was not different from zero (p = 0.756) but was smaller in children and adolescents with lower levels of motor function (p = 0.042). Ct.BMD did not differ compared to typically developing youth (p = 0.202) or between functional abilities (p = 0.168).

**Conclusions:**

Results reveal that bone and muscle size, total and cortical content, and trabecular density are impaired in children with CP and CP-like conditions; however, cortical mineralization is not impaired. Therefore, the heightened risk of fragility fractures in children and adolescents with CP and CP-like conditions is likely due to smaller and thinner bone structure. Future investigation into bone microarchitecture is warranted.

## 1 Introduction

Weight-bearing physical activity is essential for the development of bone and muscle (1, 2). Bones are particularly responsive to stimuli during childhood and adolescence because they accrue bone minerals rapidly, providing a “window of opportunity” to maximize gains from physical activity and mechanical loading (3, 4). Children with cerebral palsy (CP) and similar neuromotor impairments have reduced weight-bearing physical activity and are weaker than their typically developing peers (5, 6). Compromised bone strength significantly increases the incidence of fragility fractures in the lower limbs of individuals with CP (7–10), with an increasing incidence with age (10, 11). Further, the incidence of fragility fractures is significantly greater in those who experience more severe motor function limitations (12), and there remains a high risk of fragility fractures across all functional abilities throughout life (10). Fragility fractures cause immobilization and pain and predispose to osteoporosis, such that adults with CP have a 6–7 times greater risk of osteoporosis than individuals without CP (13, 14). Investigating the impacts of neuromotor impairments and the severity of functional limitations on bone structure during growth is crucial to understanding the factors contributing to lifelong elevated risk of fractures that cause further losses of functional abilities (15).

Individuals with CP are a heterogenous population in terms of the etiology underlying the non-progressive disturbance to brain development causing limitations in movement and posture (e.g. cerebral malformation and stroke) and the severity of the resultant functional limitations (16). Some individuals with CP walk with little to no visible impairment, whereas others have very limited voluntary movement and are unable to stand, sit, or propel a wheelchair without support. To accommodate the variability in abilities, youth with CP are classified based on their degree of motor function on the Gross Motor Function Classification System (GMFCS), with the above abilities corresponding to GMFCS levels I and V, respectively (17). As functional abilities differ across the GMFCS spectrum, so does the impact on musculoskeletal health (13, 14). Therefore, understanding bone and muscle health in childhood and adolescence across the entire spectrum is essential to help healthcare providers and clinicians select appropriate interventions to improve bone and muscle health.

While CP is the most prevalent severe childhood motor disability (18), many other conditions, such as rare genetic disorders, have similar clinical presentations impairing mobility. Most of what is known about bone and muscle development in children with neuromotor impairments comes from studies that used dual X-ray absorptiometry (DXA) to measure two-dimensional areal bone mineral density (BMD). Although the clinical gold standard, DXA cannot differentiate between cortical and trabecular bone, and outcomes are influenced by body size such that areal BMD is systematically underestimated in smaller children (19, 20). On the other hand, peripheral quantitative computed tomography (pQCT) is a three-dimensional imaging modality that assesses volumetric BMD. pQCT can also differentiate between trabecular and cortical bone and image muscle.

Few studies have examined bone and muscle health across a wide range of functional abilities. Further, the current diagnostic approach to CP leads to many uncertainties (21), resulting in gaps in knowledge about the comprehensive assessment of bone health in youth with neuromotor impairments. Therefore, this study aimed to quantify lower limb bone density, geometry, strength, and muscle cross-sectional area and density in youth with CP and other neuromotor impairments across all five GMFCS levels. We hypothesized that youth with CP have significantly impaired bone and muscle health compared to typically developing peers and that those with greater functional limitations will have greater deficits.

## 2 Materials and methods

### 2.1 Study design

This cross-sectional study invited participants from a pair of ongoing clinical trials (NCT05473676; NCT05731609) to participate. We decided to include all participants from either parent study regardless of their underlying diagnosis as they all had non-progressive neurodevelopmental conditions that impact their mobility and physical activity levels without primary impairments in bone development. The first (robotic walking) study included participants at least four years of age, unable to walk independently due to a non-progressive childhood-onset neurological impairment, who were able to fit a Trexo robotic gait trainer (<150 lbs and roughly <5’6”) and able to participate in the intensive training and assessments without medical conditions that would preclude weight-bearing or physical activity. All participants in this first parent study were GMFCS III-V. This study included participants with genetic neurodevelopmental conditions that do not align with the current definition of CP (16) (e.g. due to hypotonia being the primary motor impairment or involvement of the spinal cord). Due to the extremely rare nature of these genetic conditions and the potential that revealing the diagnosis may identify participants, the diagnosis cannot be shared and will hereafter be referred to as “CP-like”. Their bone imaging was acquired from January 2023 to June 2024. The second (power training) study included participants 8–18 years old, with CP, who were able to exert a maximum voluntary motor effort and follow directions in English who did not have an acute injury or surgery. All participants in this second parent study were GMFCS I-IV. Their bone imaging was acquired between September 2023 and June 2024.

Written informed consent was obtained from the parents or guardians of all participants, and written assent was obtained from participants. The study designs were approved by the University of Calgary Conjoint Health Research Ethics Board, REB21–1166 and REB22-1701.

### 2.2 Anthropometry

Tibia length was assessed to the nearest 0.1 cm as the distance from the medial malleolus to the tibial plateau, as described previously (22). All measurements were duplicated unless differences were >0.4 cm, where a third measurement was obtained (22). The mean of two measurements or median of three were used to calculate tibia length. Participants’ age, biological sex, diagnosis, GMFCS classification, and ethnicity were reported by parents or caregivers in questionnaires. GMFCS classification was determined by the GMFCS Family Report measure (23) following Palisano and Colleagues’ definition where those classified as level I could ambulate independently, GMFCS level III can ambulate with a handheld mobility device in some settings, and those GMFCS level V are transported in a manual wheelchair (17). In the first trial, height and weight were either reported by caregivers or through chart review. Heights were not available for all participants and were highly variable due to the severity of functional impairments. In the second community-based trial, participants’ height and weight were measured using standard techniques. Height and body mass index (BMI) Z-scores were calculated from the WHO Child Growth Standards (24).

### 2.3 Bone and muscle imaging

One of two trained operators scanned participants’ tibiae using the pQCT XCT 3000 scanner (*Stratec Medical, Pforzheim, Germany*) (25). All participants underwent pre-screening to identify previous relevant fracture/surgery history, confirm there was no implanted metal in the lower limbs, no medical devices that could be impacted by the CT scanner, no contrast imaging administered within five days of the appointment, and no chance of pregnancy. If possible, both tibiae were scanned. If there was a previous fracture or implanted metal in one distal lower limb, only the contralateral limb was scanned.

Daily single-slice quality control and monthly multi-slice quality control were performed on a hydroxyapatite calibration phantom. A two-dimensional scout view was acquired to landmark the growth plates for each participant. A reference line was placed at the most proximal end of the most distal growth plate (26) to ensure the growth plate was not scanned. From this line, scans were acquired at the 3%, 38%, and 66% sites proximal from the reference line to assess trabecular, cortical, and muscle measures, respectively. All participants were scanned using the manufacturer’s protocol of 2.4 mm slice at 400 μm, tube voltage of 46 kV DC and current of <0.3 mA. The scout view duration was ~80 seconds, and the slice duration was ~100 seconds (30 mm/sec). The effective dose for this protocol was less than 3 μSV per series of scans. The total scan measurement time was ~8 minutes. In the instance of motion artifact, scans were repeated once. *In vivo* precision at the sites used in this study is excellent (<2% CV) (27).

A single researcher analyzed all scans and evaluated all scans for motion artifacts using a scale from 1 (no motion) to 5 (substantial motion) (28). All scans with a grade of 3 or below were analyzed (software version 6.20); scans graded as 4 were reviewed by a second researcher to confirm grading and exclusion. Analysis included one leg per participant. In participants with bilateral impairments, the limb with the best quality scan was used. In participants with unilateral impairments, the scan of the impaired limb was included in the analysis if possible. In recognition that physiological differences are present in the side not noted to have motor impairments in people with unilateral CP, the less impaired limb was included in the analysis if it was the only scan available.

The region of interest was manually selected for each scan. In each scan, a density threshold differentiated between cortical and trabecular bone, muscle, fat, and other *in vivo* tissues. The 3% total bone mineral content (BMC) and trabecular BMD (Tb.BMD) measurements were calculated using a threshold of 180 mg/cm^3^ and a cortical threshold of 480 mg/cm^3^, with peel mode and contour mode “1” and a trabecular area of 45%. For the 38% slice measurements, cortical measures including cortical BMD (Ct.BMD), cortical BMC (Ct.BMC), cortical area (Ct.Ar), cortical thickness (Ct.Th) and endosteal and periosteal circumference were analyzed with a threshold of 710 mg/cm^3^, with peel mode and contour mode “1”, and a trabecular area of 45%. Strength measures, including section modulus (Z) and polar stress-strain index (SSI_p_), were calculated with a threshold of 480 mg/cm^3^. In brief, Z represents a measure of bone strength by taking into account bone size, while SSI_p_ is a density-weighted measure that considers bone size and material composition to provide estimates of bending strength (29). The muscle measurements, muscle cross-sectional area (MCSA) and density were calculated with a threshold of 280 mg/cm^3^ with contour mode “1”, peel mode “2”, and smoothing filter F0305. Muscle separation from subcutaneous fat utilized a threshold-based edge detection with a threshold of 40 mg/cm^3^ with contour mode “3”, peel mode “1”, and smoothing filter F030505; a manual ROI was drawn around the muscle (30). To determine MCSA, bone area was subtracted from total bone and muscle area combined. Muscle density was calculated by dividing total muscle mass by MCSA. Measurements by slice at the 3% site include total BMC (mg/mm) and Tb.BMD (mg/cm^3^); at the 38% slice include Ct.BMD (mg/cm^3^), Ct.BMC (mg/mm), Ct.Th (mm), Ct.Ar (mm^2^), endosteal circumference and periosteal circumference (mm), Z (mm^3^) and SSI_p_ (mm^3^); and at the 66% slice include MCSA (mm^2^) and muscle density (mg/cm^3^). All values (excluding SSI_p_ and muscle density) were converted to age-, sex-, and ethnicity-matched Z-scores from a normative population (31). All participants were matched to “White” normative data unless they identified as “Black” to align with reference data.

### 2.4 Positioning in the scanner

**Figure 1:**
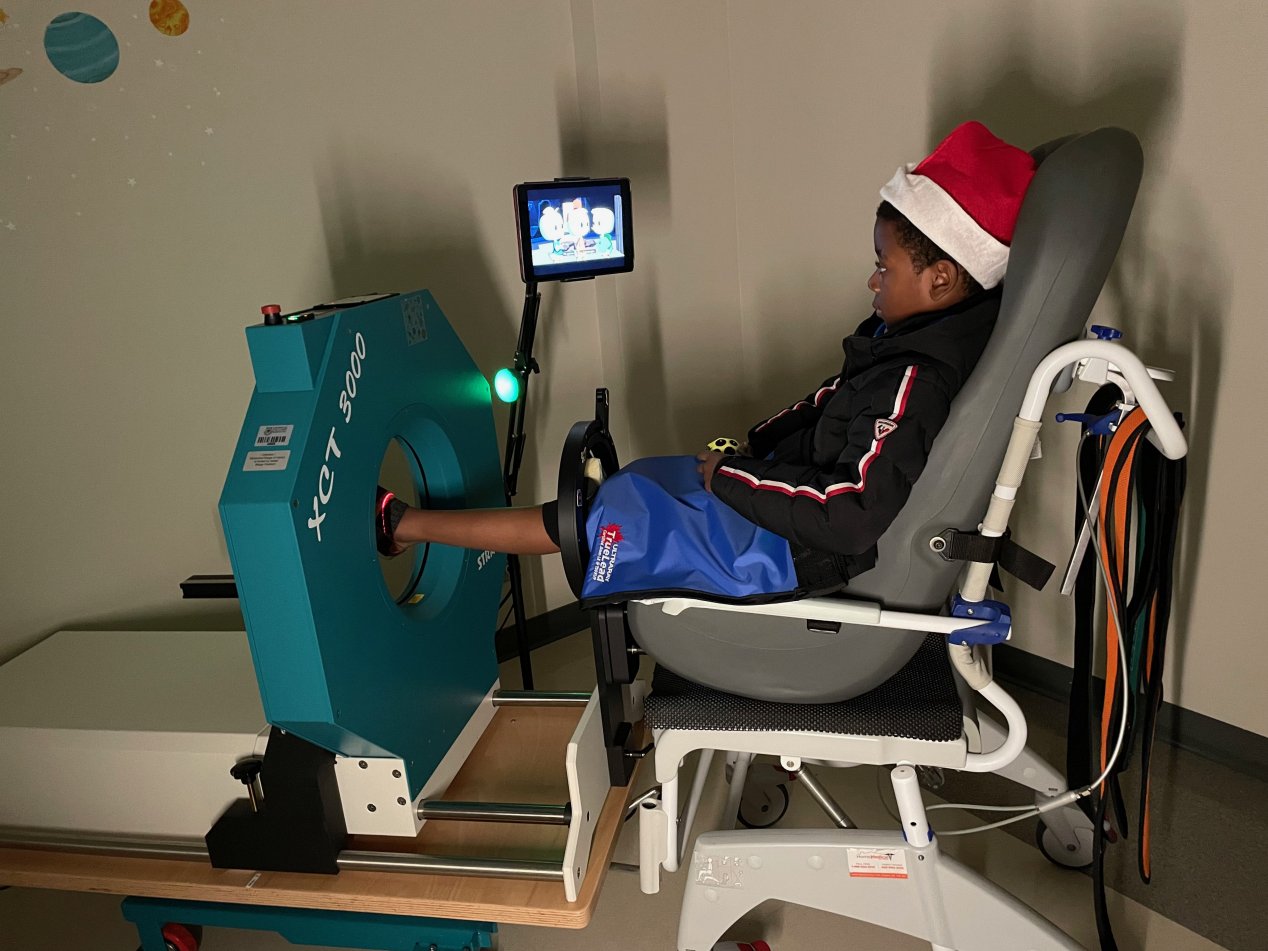
Participant positioned in the pQCT scanner.

Positioning children with neuromotor impairments in the scanner can be challenging (32). The presence of metal in the legs and the short stature of younger participants made acquiring scans on both legs or at the 66% slice (muscle assessment) particularly difficult (66% slice not acquired in 7 participants). Further, as involuntary movement is common in individuals with CP and CP-like conditions, having participants sit stationary for the ~8-minute scan proved difficult. We adopted several modifications over the first months to increase the success of imaging. For example, the use of adapted seating, a Special Tomato Soft-Touch Sitter (*Bergeron Health Care, Dolgeville New York*), aided in positioning children and youth comfortably by adding a slight recline to better align their legs with the scanner and avoid stretching the frequently spastic hamstrings (**Figure 1**). Further, dimming the lights in the room, having a movie/show on a device that was fixed and out of reach, holding a stuffed animal, and placing stickers on the wall to look at helped participants remain calm and still for the scan duration. Lastly, before the appointment, we sent families a video of what to expect during the appointment, including the sounds the machine makes to reduce anxiety.

### 2.5 Statistical analysis

Data were assessed for normality through exploratory histograms and boxplots. The median and interquartile range as well as mean and standard deviations are reported for continuous variables and percent for categorical variables. One-sided Wilcoxon sign rank tests determined whether Z-scores were significantly different from zero (expected Z-scores for typically developing peers). Mann-Whitney tests determined whether variables differed between those with lower functional abilities (GMFCS III-V) compared to those with greater functional abilities (GMFCS I-II), with significance set at p<0.05.

## 3 Results

Twenty-six participants aged 4–17 years consented to bone imaging. Twenty-three participants were diagnosed with CP, and three participants were diagnosed with other non-progressive neuromotor impairments (CP-like). Twenty-two participants had valid scans from at least one impaired leg and were included in the analysis; twenty were diagnosed with CP, and two were diagnosed with CP-like conditions. Participant characteristics are described in **Table 1**. Amongst those with a diagnosis of CP, 75% had bilateral impairments, and both participants with CP-like conditions had bilateral impairments. All participants with CP had spasticity (GMFCS I-V, n=15); four had a mixed motor impairment (e.g. spastic and either dyskinetic (GMFCS IV, n=3) or ataxic motor impairment (GMFCS II, n=1); one had dystonic spasticity (GMFCS II, n=1). Both CP-like participants had neurodevelopmental disorders causing hypotonia (GMFCS IV, n=2) and like participants with CP, neither had a metabolic or other disorder associated with primary impairments in bone.

**Table 1:** Overview of participant characteristics. Mean (+/- SD) and median (Interquartile range), unless otherwise indicated.

	Variable (n=22)	Mean (+/- SD)	Median (IQR)
Population	Age (Years)	11.8 (3.6)	12.2 (9.2, 14.8)
	Sex (M:F)	17:5	
	*Height (cm)	145.7 (22.0)	144.0 (130.4, 167.0)
	*Height Z-score	-0.78 (1.46)	-0.69 (-1.94, 0.76)
	*Weight (kg)	38.7 (20.4)	31.2 (22.6, 56.7)
	*BMI (kg/m^2^)	19.3 (5.2)	16.8 (16.2, 23.7)
	*BMI Z-score	0.03 (1.25)	0.05 (-0.71, 0.88)
	GMFCS Level (I/II/III/IV/V)	5:7:3:6:1	
	Motor Impairment Type (Spastic/Mixed-Spastic-Dyskinetic/Mixed-Spastic-Ataxic/Spastic-Dystonic/Hypotonia)	15:3:1:1:2	
	Tibia Length (mm)	320 (70)	315 (269, 385)
3%Slice	Total BMC (mg/mm)	187.1 (108.5)	158.4 (101.9, 277.4)
	Tb.BMD (mg/cm^3^)	178.3 (48.4)	179.7 (141.0, 213.3)
38% Slice	Ct.BMC (mg/mm)	178.2 (81.2)	175.4 (103.6, 235.2)
	Ct.BMD (mg/cm^3^)	1100.7 (60.4)	1096.1 (1077.2, 1129.3)
	Ct.Th (mm)	3.5 (0.9)	3.5 (2.7, 4.1)
	Ct.Ar (mm^2^)	161.4 (78.2)	155.5 (94.4, 211.5)
	Periosteal Circumference (mm)	55.0 (14.2)	54.1 (41.4, 65.3)
	Endosteal Circumference (mm)	33.2 (9.7)	32.9 (25.4, 37.9)
	Z (mm^3^)	849 (599)	702 (327, 1215)
	SSI_p_ (mm^3^)	1104 (768)	884 (432, 1524)
66% Slice	MCSA (mm^2^)	3633 (1638)	3587 (2574, 4467)
	Muscle Density (mg/cm^3^)	76.4 (3.8)	77.4 (74.2, 79.1)

The 3, 38, and 66% slices included n=20, n=20, and n=17 participants, respectively. *n=17 for height and BMI, n=22 for weight.

### 3.1 Participant characteristics

Participants included all five GMFCS levels (I:23%; II:32%; III:14%; IV:27%; V:4%). Most participants were biologically male (77%), and 65% identified as White, 14% Black, 9% Indigenous, 4% Arab, 4% Latin American, and 4% Mixed population groups. Height and BMI Z-scores ranged from -3.09 to 2.33 and were not statistically different from zero (p=0.071 and p=0.926, respectively). Tibia length Z-scores ranged from -5.49 to 1.64 (median: -1.05), were significantly different from zero (p=0.019), and were lower in those with greater functional limitations (p=0.015).

### 3.2 pQCT imaging

Of one hundred forty-eight scans acquired from 26 participants, 47 scans from GMFCS II-IV were excluded due to excessive motion artifact (grade of >3). Four participants GMFCS levels III (n=1) and IV (n=3) had all scans excluded (i.e. no valid scans at any site for either leg). Out of all scans acquired, 68% were used in this analysis. Amongst those GMFCS levels I-II, 75%, 85%, and 92% of scans were of acceptable quality for the 3%, 38%, and 66% slices, respectively. GMFCS levels III-V had lower quality scans, with 45%, 48%, and 71% of scans of acceptable quality at the 3%, 38%, and 66% slices, respectively.

### 3.3 Bone outcomes

#### 3.3.1 Trabecular bone

**Figure 2:**
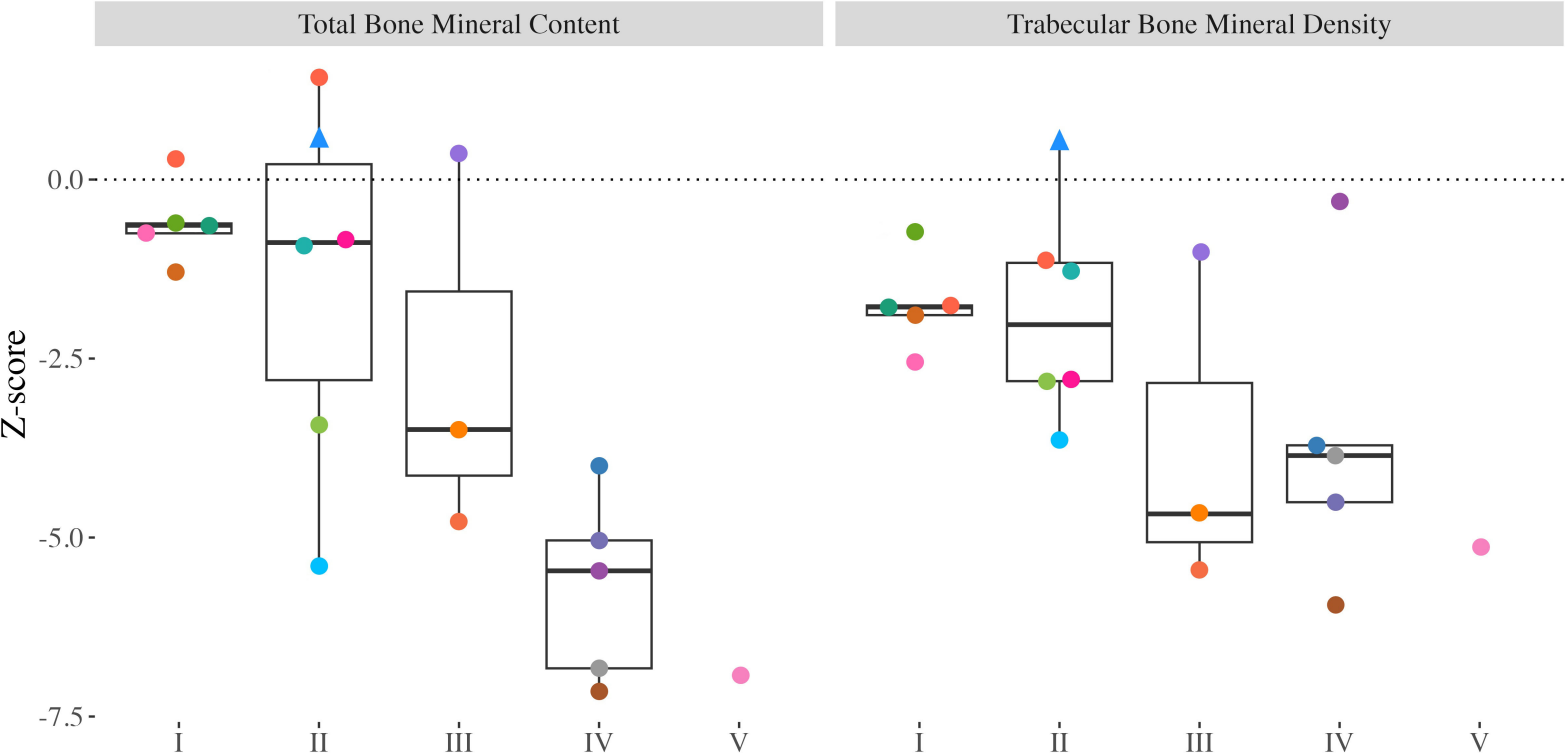
Distal tibia Z-scores from all participants with valid scans at the 3% slice. Individual dots represent a unique participant, colours correspond to the same participants across **Figures 2**-**4**. Triangles indicate the less impaired leg is included in the analysis (n=1). Bone mineral content (BMC) and trabecular bone mineral density (Tb.BMD).

Total BMC and Tb.BMD Z-scores ranged from -7.15 to 1.43 (median: -2.36) and -5.94 to 0.52 (median: -2.66), respectively. Both total BMC and Tb.BMD were significantly different from zero (**Figure 2**; p<0.001, for both). Total BMC Z-scores were significantly lower in youth with CP and CP-like conditions with greater functional limitations (GMFCS I-II *vs*. III-V; p<0.001). A trend for lower Tb.BMD was observed in those with greater functional limitations (p=0.076).

#### 3.3.2 Cortical bone and bone strength

**Figure 3:**
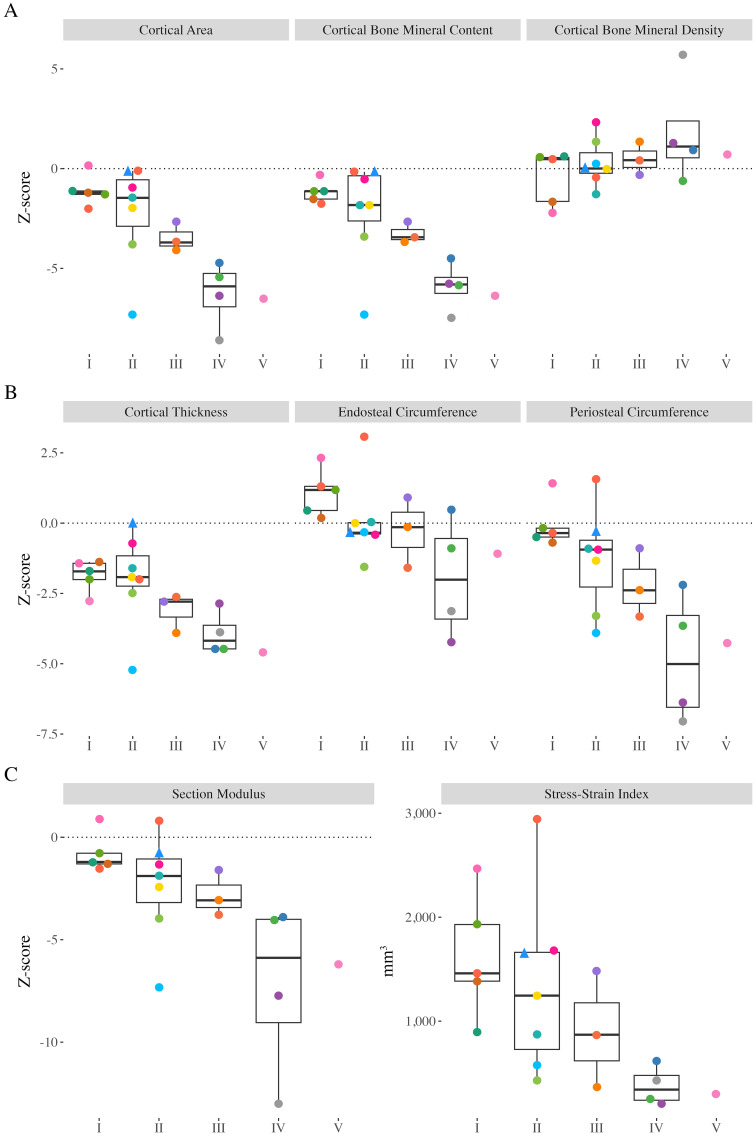
Distal tibia Z-scores from all participants with valid scans at the 38% slice. Individual dots represent a unique participant, colours correspond to the same participants across **Figures 2**-**4**. Triangles indicate the less impaired leg is included in the analysis (n=1). **(A)** Cortical area (Ct.Ar), cortical bone mineral content (Ct.BMC), cortical bone mineral density (Ct.BMD). **(B)** Cortical thickness (Ct.Th), endosteal circumference, periosteal circumference. **(C)** Section modulus (Z) and polar stress-strain index (SSI_p_).

Ct.BMC and Ct.Th Z-scores ranged from -7.47 to -0.16 (median: -2.25) and -5.20 to -0.01 (median: -2.55), respectively, and were significantly different from zero (**Figure 3**; p<0.001). Children and adolescents with greater motor function limitations had lower Ct.BMC (GMFCS I-II *vs*. III-V; p=0.002) and Ct.Th (p=0.004) compared with those with greater functional abilities. Ct.Ar and periosteal circumference Z-scores ranged from -8.58 to 0.17 (median -2.33) and -7.06 to 1.55 (median: -1.09), respectively, were significantly different from zero (p<0.001; **Figure 3**), and significantly lower in those with greater functional limitations (p<0.001 and p=0.002, respectively). Endosteal circumference Z-scores ranged from -4.23 to 3.07 (median: -0.07) and did not differ compared to typically developing peers (p=0.756). However, endosteal circumference was significantly lower in those with greater functional limitations (p=0.042). Ct.BMD Z-scores ranged from -2.21 to 5.71 (median: 0.45), were not significantly different from zero (p=0.202), and did not differ between children and adolescents of varying functional abilities (p=0.168). The Z Z-scores ranged from -13.00 to 0.90 (median: -2.14), were significantly lower than typically developing peers (p<0.001), and were significantly lower in those with greater functional limitations (p<0.001). SSI_p_ ranged between 206 to 2947 mm^3^ (median: 884) and were significantly lower in children and adolescents with greater functional limitations (p=0.002).

#### 3.3.3 Muscle outcomes

**Figure 4:**
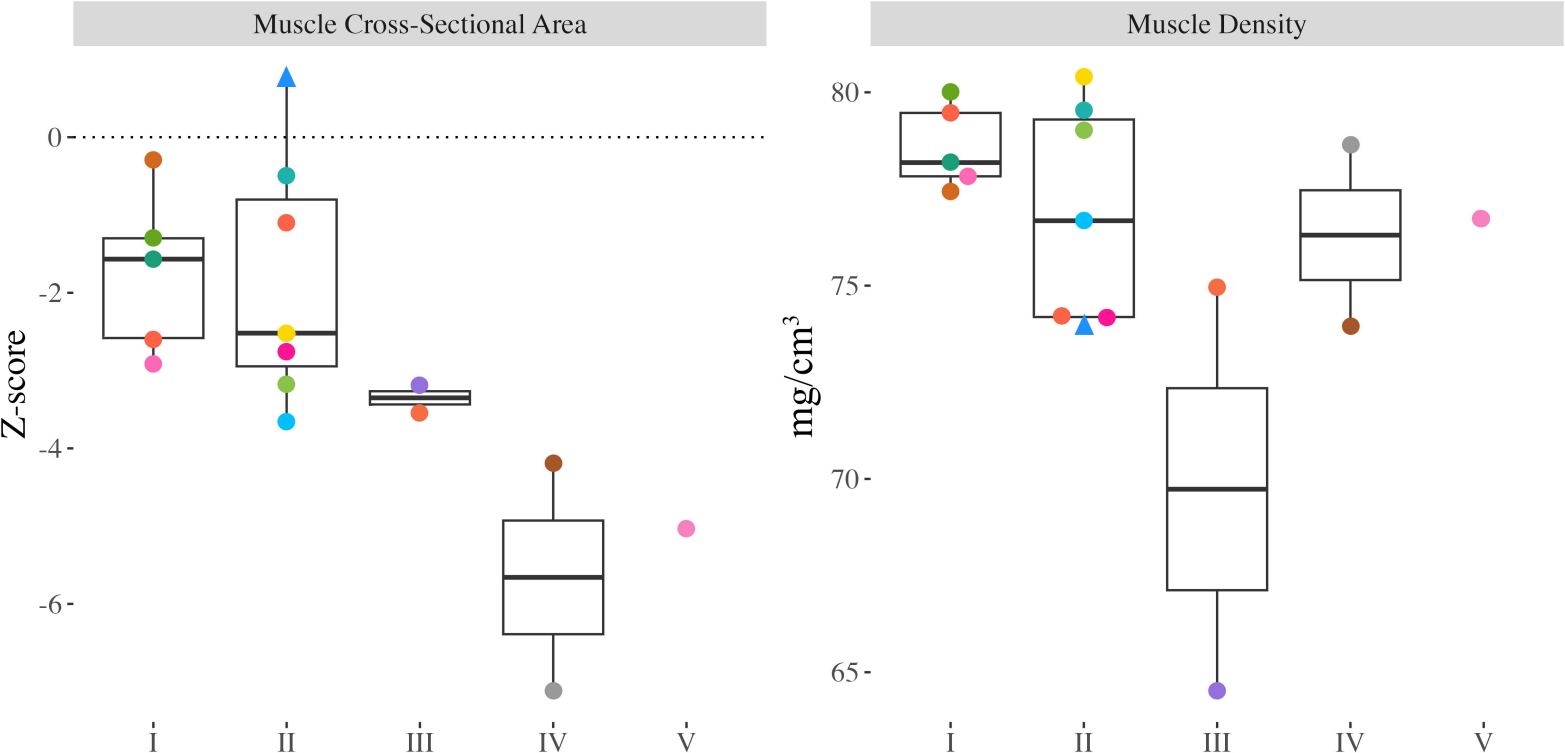
Proximal tibia muscle cross-sectional area (MCSA) Z-scores and muscle density from all participants with valid scans at the 66% slice. Individual dots represent a unique participant, colours correspond to the same participants across **Figures 2**-**4**. Triangles indicate the less impaired leg is included in the analysis (n=1).

MCSA Z-scores ranged from -7.12 to 0.78 (median: -2.73), were significantly different from zero (**Figure 4**; p<0.001), and were significantly lower in children and adolescents with greater functional limitations (GMFCS I-II *vs*. III-V; p=0.003). Muscle density did not differ between children and adolescents with varying functional abilities (p=0.676).

## 4 Discussion

This study quantifies bone and muscle health at the tibia in youth with CP and CP-like conditions using pQCT and reveals significant deficits compared to age-, sex-, and ethnicity-matched normative data. Across all GMFCS levels, children with CP and CP-like conditions exhibit significantly lower tibial bone and muscle measures, including total and cortical BMC, cortical size, strength and MCSA, with deficits correlating with functional limitations. Our findings support the notion that elevated lower-limb fracture risk in children and adolescents living with CP can be partially attributed to their slender, thinner, long bone structure. As bone strength in bending is proportional to bone area (bending strength is equal to the cube of the bone’s diameter) (33), smaller-diameter bones that are thinner and have lower mineral content are less able to resist fracture (32–36).

Since bones adapt to mechanical loads, ambulation is essential for bone development, stimulating bone accrual and mineralization (1, 2, 37). The deficits we observed in bone mineral content, size, and strength were exacerbated in children and adolescents who experience lower levels of motor function. The early onset of limited loading experienced by individuals with CP and CP-like conditions is likely an important factor contributing to their lower total and cortical bone mineral content (38) accrual. Our findings regarding lower total and cortical bone content align with previous work demonstrating lower values compared to typically developing peers and larger deficits with greater functional limitations (34). In our cohort, Tb.BMD at the distal tibia was significantly lower than that of a typically developing population and, although not statistically significant, showed a trend consistent with previous literature suggesting declines with increasing functional limitations (35). The significantly lower trabecular BMD may suggest that youth with CP and CP-like conditions have impaired trabecular mineralization or fewer and thinner trabeculae (34).

In contrast to other cortical measures, cortical BMD did not differ from a typically developing population or across diagnosis severity. Our finding of average cortical BMD for age aligns with earlier reports showing no differences from typically developing peers (32). Cortical BMD results were unique because there was no association with functional abilities, which likely reflects the proportional relationship between lower cortical content and smaller bone area. Previous studies using DXA indicated impaired mineralization in children with CP who are unable to ambulate independently (39). For example, distal femur DXA scans revealed that absolute areal BMD increased slower than expected for typically developing peers (39). However, previous findings of impaired mineralization (39) may be explained by the fact that DXA systematically underestimates areal BMD in smaller bodies (20), as in this population, and cannot separate trabecular and cortical bone compartments. Future research utilizing high-resolution modalities such as HR-pQCT may provide better insights into trabecular and cortical microarchitecture, including cortical porosity and trabecular separation, that contribute to BMD measurements and enhance our understanding of bone strength in children and youth with CP and CP-like conditions.

Children with CP and CP-like conditions who experience limited ambulation and loading have greater skeletal fragility (10, 35, 36, 40, 41). Given the importance of loading for periosteal expansion, it was not surprising that we observed more substantial deficits in periosteal expansion and cortical area in youth with greater functional limitations. The smaller periosteal circumference observed in our cohort aligns with previous literature (32) and may be attributed to the early onset of limited loading, which impairs periosteal expansion regardless of GMFCS classification (5, 6). A previous study using MRI highlighted that ambulation is essential for endosteal and periosteal expansion at the femoral shaft in children classified as GMFCS III-V (5). Although we did not observe lower endosteal circumferences in youth classified as GMFCS I-V compared to a typically developing population, we found that children and adolescents who experience lower levels of motor function had smaller endosteal circumferences. Our observations suggest that endosteal circumference at the tibial shaft is not compromised in individuals with greater functional abilities and that even partial loading may positively influence endocortical bone modelling. Collectively, reduced cortical bone content and size contributed to significantly lower bone strength in children and adolescents with CP and CP-like conditions. Deficits in tibial bone strength were evident across all GMFCS levels and increased with increasing functional limitations. Our finding that section modulus (Z) was 48% lower than expected compared to a normative population aligns with previous research (6) and corroborates greater deficits in strength with increasing functional limitations. Although we speculate that reduced ambulation may be the primary cause of lower bone strength in youth with CP and CP-like conditions, we acknowledge that other factors, such as gait abnormalities that are common in this population (42) as well as nutritional deficiencies due to feeding difficulties (7), may also contribute to reduced bone size and strength. Altered gait would simultaneously change the loading experienced by bones (43) and subsequently affect adaptive responses. Similarly, inadequate nutrition would limit growth, reducing bone size and strength (39). The interplay of limited loading and gait abnormalities resulting in compromised bone size and strength in children with CP and CP-like conditions likely explains their higher incidence of lower limb fragility fractures (6).

To better understand how bone development differs in children with CP and CP-like conditions, the influence of muscle should also be considered. Muscle size can be used as a proxy for the internal muscle strains exerted on bones (36, 44). We found that children with CP and CP-like conditions who experience lower levels of motor function exhibited significantly smaller MCSA (approximately 49% lower than normative data), corroborating similar findings from previous studies (45, 46). Substantially lower MCSA likely reflects a lack of loading and contributes to our observed deficits in bone accrual and strength. We also assessed muscle density, a proxy for muscle fat content (29). We expected to find greater fatty infiltration (lower muscle density) in children and adolescents who experience lower levels of motor function since fatty infiltration is inversely related to physical activity levels (6, 29, 47); however, we did not see differences in muscle density across GMFCS levels (6, 36). As there are no reference values for muscle density, we could not assess whether muscle density differed compared to a typically developing population.

## 5 Limitations

We note several limitations of our study. Although current results are compared to a normative population of typically developing youth matched for age, sex, and ethnicity, our small sample of children and adolescents with CP may not be representative of the general population of youth living with CP. We included the scan from the less impaired leg of one participant with unilateral CP due to a metal implant in the more impaired leg. This may have underestimated the impact on musculoskeletal outcomes. Further, because we improved our methods incrementally and started with participants with higher GMFCS levels, these participants may be underrepresented. We did not assess other factors that can impact bone development in participants, including vitamin D or calcium levels, which were previously found to be lower in children with CP and CP-like conditions and impact bone (48, 49), anticonvulsant use (48), hormone status (2), or nutritional status (7). We included two participants with CP-like conditions; however, their hypotonia may have influenced bone accrual differently than those with CP. Further, the available normative data were for White and Black youth; therefore, we could not adjust for other ethnicities and Z-scores may not accurately account for ethnic differences in bone strength (50). Future work would benefit from a more representative normative data set as well as exploration into biomarkers of bone turnover in youth with CP and CP-like conditions. Finally, a higher resolution modality in future, such as HR-pQCT, would provide valuable information regarding trabecular microarchitecture.

## 6 Conclusions

This study highlights significant deficits in bone and muscle health in youth with CP and CP-like conditions, especially in youth with lower functional abilities. Smaller and thinner bones may make children and adolescents with CP and CP-like conditions more susceptible to fragility fractures in the lower limbs. Despite lower bone mineral content and limited periosteal expansion, cortical bone mineralization was not impaired in youth with CP and CP-like conditions. Knowing these substantial deficits, interventions that improve bone and muscle strength are warranted to reduce fragility fractures and lifelong comorbidities for youth with CP and CP-like conditions.

## Data Availability

Some of the data that support the findings of this study are openly available in Bone Bank: A Bone Health Database for Children with Cerebral Palsy and Neuromotor Impairments at https://doi.org/10.5683/SP3/VLV4YC. Due to the nature of the research and participant privacy, some supporting data is only available upon request with research ethics board approval.
